# Danger, Danger, High Output

**DOI:** 10.1016/j.jscai.2023.101122

**Published:** 2023-08-22

**Authors:** Cooper B. Kersey, Barbara A. Danek, Christine J. Chung

**Affiliations:** Division of Cardiology, University of Washington, Seattle, Washington

**Keywords:** heart failure, high-output heart failure, transthoracic echocardiogram

## Case

A 42-year-old man presented with subacute progressive dyspnea on exertion, 3-pillow orthopnea, abdominal distension, and bilateral lower extremity edema. His medical history was notable for mantle cell lymphoma treated with chemotherapy alone and in remission. He had been diagnosed with idiopathic constrictive pericarditis 3 months prior to presentation based on a chest computed tomography demonstrating pericardial thickening and transthoracic echocardiogram (TTE) findings of annulus reversus, ventricular septal shift, and a plethoric inferior vena cava. He was treated with anterior phrenic nerve to phrenic nerve pericardiectomy. At the time of his current presentation, his physical examination was notable for a jugular venous pressure of 15 cm H_2_O, abdominal distension without tenderness, and 2+ bilateral lower extremity edema.

**Figure 1:**
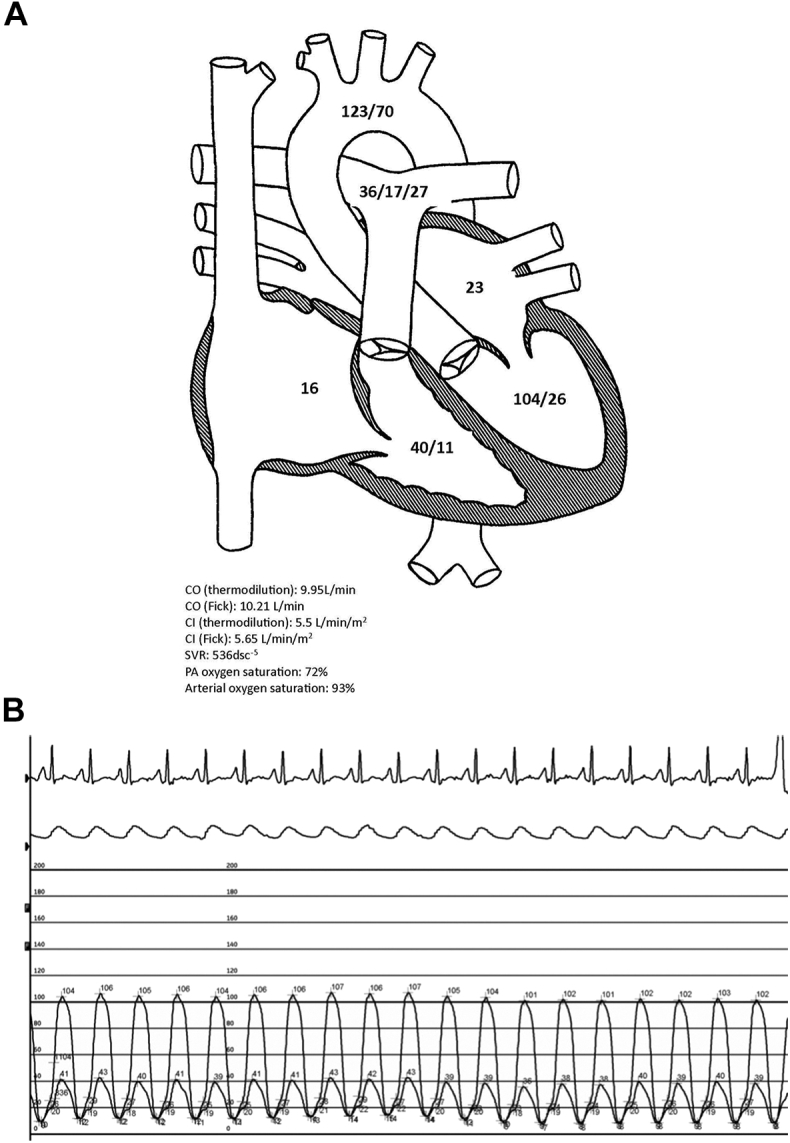
**Findings of simultaneous right and left heart catheterization.** (**A**) The patient’s hemodynamics as measured by right heart catheterization. (**B**) Simultaneous left and right heart catheterization without respiratory variation of the left or right ventricular systolic pressures. This argues against constrictive or restrictive physiology as the cause of the patient’s symptoms. CI, cardiac index; CO, cardiac output; PA, pulmonary artery; SVR, systemic vascular resistance.

A TTE revealed normal biventricular systolic function, no significant valvular abnormalities, an estimated right atrial (RA) pressure of 15 to 20 mm Hg, mildly elevated estimated pulmonary artery systolic pressure of 38 to 43 mm Hg, and a high stroke volume of 103 mL (significantly increased from 51.4 mL 3 months prior). The differential diagnosis for the patient’s persistent volume overload following pericardiectomy included residual constrictive pericarditis from his posterior pericardium, restrictive cardiomyopathy, right ventricular failure after pericardiectomy, and noncardiac causes of volume overload, such as cirrhosis or nephrosis. The patient underwent simultaneous right and left heart catheterization (Table), which revealed mildly elevated biventricular filling pressures (RA pressure of 16 mm Hg and pulmonary capillary wedge pressure of 23 mm Hg), elevated cardiac output (10.2 L/min by Fick and 10 L/min by thermodilution) and cardiac index (5.6 L/min/m^2^ by Fick and 5.5 L/min/m^2^ by thermodilution), and low systemic vascular resistance (536 dsc^−5^) (Figure 1A). There was no significant respirophasic variation in left ventricular pressure (Figure 1B). The etiology of high-output heart failure was sought, and the patient was found to have cirrhosis on a right upper quadrant ultrasound. There had been no prior physical examination or abnormal laboratory findings to suggest that the patient had cirrhosis. Six months prior to his initial diagnosis of constrictive pericarditis, the patient had undergone a liver biopsy as part of the workup of his lower extremity edema, which did not show any evidence of cirrhosis. Because the patient had developed cirrhosis in the interval 8 months from the time of initial diagnostic evaluation, it was felt that the cirrhosis was due to hepatic congestion from his constrictive physiology that went undiagnosed for several months. He underwent diuresis and experienced improvement in symptoms.

**Table 1:** Hemodynamics from simultaneous right and left heart catheterization

Hemodynamic parameter	Value on right and left heart catheterization
Right atrial pressure, mm Hg	16
Right ventricular pressure, mm Hg	40/11
Pulmonary artery pressure, mm Hg	36/17/27
Pulmonary capillary wedge pressure, mm Hg	23
Left ventricular pressure, mm Hg	104/26
Aortic pressure, mm Hg	123/70
Arterial oxygen saturation, %	93
Pulmonary artery oxygen saturation, %	72
Cardiac output (TD), L/min	9.95
Cardiac output (Fick), L/min	10.21
Cardiac index (TD), L/min/m^2^	5.50
Cardiac index (Fick), L/min/m^2^	5.65
Systemic vascular resistance, dsc^−5^	536

TD, thermodilution.

## Discussion

High-output heart failure is the clinical syndrome of heart failure in a patient with high cardiac output due to an underlying vasodilatory or hypermetabolic state.^1^ The most common causes of high-output heart failure in the modern era are obesity (33%), liver disease (23%), arteriovenous shunt (22%), pulmonary disease (16%), and myeloproliferative disease (8%).^2^ High-output heart failure can be challenging to diagnose with noninvasive modalities given that stroke volume and cardiac index are not routinely reported for all TTEs. To make the diagnosis by echocardiogram requires enough clinical suspicion for high-output heart failure to prompt the reading echocardiographer to highlight the stroke volume and/or calculate the cardiac index using the left ventricular outflow tract velocity time integral. Our patient had been hospitalized for 2 months and had undergone several TTEs without an explanation for his symptoms before he was referred for an invasive hemodynamic assessment (although his stroke volume had increased by echocardiographic assessment). Right heart catheterization in patients with high-output heart failure will demonstrate a characteristic hemodynamic profile: elevated cardiac index, low systemic vascular resistance, and elevated filling pressures. A single-center retrospective study of 120 patients with high-output heart failure found a mean RA pressure of 11 mm Hg, a mean pulmonary artery systolic pressure of 37 mm Hg, a mean pulmonary capillary wedge pressure of 18 mm Hg, a cardiac index of 5.7 L/min/m^2^, and a mean systemic vascular resistance of 682 dsc^−5^.^2^ Prompt diagnosis is imperative because definitive treatment requires addressing the underlying cause and mortality is high if left untreated.^1,2^ This case illustrates the importance of invasive hemodynamic assessment of unexplained heart failure.

## Pearls in Hemodynamics from editors Larry S. Dean, MD, and Morton J. Kern, MD

1. Common to constriction, restriction, and high-output failure are elevated right and left heart filling pressures.
2. Respirophasic variation in right and left heart pressures is helpful in differentiating constriction from restriction but is not seen in high-output failure.
3. Invasive measurement of cardiac output/index differentiates high-output failure from the more common forms of failure due to constrictive or restrictive pathology.
4. Incomplete assessment of complex physiology can lead to inappropriate patient management decisions.
